# Direct Effects of Toxic Divalent Cations on Contractile Proteins with Implications for the Heart: Unraveling Mechanisms of Dysfunction

**DOI:** 10.3390/ijms241310579

**Published:** 2023-06-24

**Authors:** Oksana P. Gerzen, Veronika O. Votinova, Iulia K. Potoskueva, Alyona E. Tzybina, Larisa V. Nikitina

**Affiliations:** Institute of Immunology and Physiology of the Ural Branch of the Russian Academy of Sciences, 620049 Ekaterinburg, Russiatzybina.elena2013@yandex.ru (A.E.T.);

**Keywords:** cardiotoxicity, toxic ions, divalent cations, heart muscle, EF-hand proteins, myosin, regulatory light chain, troponin C, ATPase activity

## Abstract

The binding of calcium and magnesium ions to proteins is crucial for regulating heart contraction. However, other divalent cations, including xenobiotics, can accumulate in the myocardium and enter cardiomyocytes, where they can bind to proteins. In this article, we summarized the impact of these cations on myosin ATPase activity and EF-hand proteins, with special attention given to toxic cations. Optimal binding to EF-hand proteins occurs at an ionic radius close to that of Mg^2+^ and Ca^2+^. In skeletal Troponin C, Cd^2+^, Sr^2+^, Pb^2+^, Mn^2+^, Co^2+^, Ni^2+^, Ba^2+^, Mg^2+^, Zn^2+^, and trivalent lanthanides can substitute for Ca^2+^. As myosin ATPase is not a specific MgATPase, Ca^2+^, Fe^2+^, Mn^2+^, Ni^2+^, and Sr^2+^ could support myosin ATPase activity. On the other hand, Zn^2+^ and Cu^2^ significantly inhibit ATPase activity. The affinity to various divalent cations depends on certain proteins or their isoforms and can alter with amino acid substitution and post-translational modification. Cardiac EF-hand proteins and the myosin ATP-binding pocket are potential molecular targets for toxic cations, which could significantly alter the mechanical characteristics of the heart muscle at the molecular level.

## 1. Introduction

Heart contraction is a highly coordinated and complex process that involves the interplay of thin and thick filaments, which slide one past another with no alteration in their length [1]. The thick filament primarily consists of myosin molecules, which contain two heavy and four light chains, including two calcium-binding regulatory and two non-calcium-binding essential light chains [2]. The thin filament comprises actin, tropomyosin, and troponin subunits T, I, and C [3].

The concentration of intracellular calcium plays a crucial role in regulating heart contraction by binding calcium ions to the troponin C subunit and altering the structure of troponin [4]. This causes the tropomyosin strands to move across the actin filament, opening sites for binding with myosin heads—the N-terminus of myosin heavy chains [5]. The energy required for myosin to propel thin filaments is provided by ATP hydrolysis [6] in the myosin ATP-binding pocket, which can also bind magnesium ions [7].

In summary, heart contraction involves multiple proteins, including calcium- and magnesium-binding protein structures, viz. troponin C, the myosin regulatory light chain, and the ATP-binding pocket of myosin [8,9,10]. Calcium and magnesium are involved in a complex cascade of protein interactions during heart contraction, in which timing and coordination are highly important for the entire process. Therefore, calcium and magnesium ions play an essential role in regulating actomyosin interaction during heart contraction. However, is it possible for other cations to intervene in this well-orchestrated process?

## 2. Accumulation, Competition, and Toxicity of Divalent Cations

### 2.1. Accumulation in the Myocardium and Cardiotoxic Effect of Divalent Cations

Myocardium was found to accumulate various chemicals, such as aluminium [11,12,13,14], antimony [15], barium [11], cadmium [11,16,17,18,19,20,21,22,23,24,25,26], calcium [27,28,29], caesium [11], cobalt [15,18], copper [11,18,27,28,29,30,31,32], chromium [11,15,27,33], gold [15], iron [27,28,34], lead [11,18,19,22,35], magnesium [28,29], manganese [11,18,27,28,36], mercury [15,16], molybdenum [11,28], nickel [11,18,27,33,34,37,38], strontium [11,27], tin [11], and zinc [11,17,18,19,20,22,27,28,29,32]. Amongst them, there are elements with the primary oxidation state of +2.

The above-mentioned divalent cations can be divided into three groups [39,40,41]:

1. Essential ions for physiological processes (Ca^2+^, Cu^2+^, Fe^2+^, Mg^2+^, Zn^2+^, Mo^2+^).
2. Nonessential ions with no or low or unknown toxicity (Ba^2+^, Sr^2+^, Co^2+^, Ni^2+^).
3. Nonessential and highly toxic ions (Cd^2+^, Pb^2+^, Hg^2+^).

Some other less toxic mono-, di-, and trivalent cations also tend to accumulate in the myocardium and have been used as calcium analogues in various studies, e.g., La^3+^ and Tb^3+^ have been widely used as Ca^2+^ surrogates to examine metal binding properties and conformational changes of Troponin C [39,40,42].

Nevertheless, we focused on the nonessential toxic divalent cations from the 3rd group viz. Cd^2+^, Pb^2+^, and Hg^2+^, which are ubiquitous chemicals and considered to be important co-exposures [43]. Chronic exposure to cadmium [44,45], lead [46,47,48], and mercury [49] has been associated with multiple cardiovascular diseases. Cardiotoxicity after chronic and acute exposure was experimentally confirmed in the animal models for cadmium [16,50,51,52,53,54], lead [19,35,55,56,57], and mercury [16]. For each of viz. cadmium [58,59,60,61], lead [40,62,63], and mercury [40,64,65], the direct cardiotoxic effect was confirmed at different levels of organization, including the whole heart, multicellular, cellular, and protein level.

It is worth noting that excess of some essential divalent cations from the 1st and 2nd groups could also be highly toxic. For example, Cu^2+^ could have mediated toxic effects through chronic exposure [31] and direct toxic effects [66,67,68] on the cardiovascular system. Ni^2+^ [69,70,71] and V^2+^ [69,71] exposure could also be connected with cardiovascular diseases (CVD). Co-exposure to Cu^2+^, Mo^2+^ and V^2+^ could be closely connected with CVD mortality [72]. Other essential cations like Ba^2+^, Mn^2+^, Co^2+^, Ni^2+^, and Zn^2+^ could have a direct effect on cardiac cells [61,73], especially on transport channels, whilst Ca^2+^, Mg^2+^, and Sr^2+^ appear to have less of an effect [73].

There have been informative reviews that broadly cover the topic of the effect of exposure to toxic metals on the cardiovascular system. For instance, there are reviews available for xenobiotic metals like lead [74,75], cadmium [74,75,76,77], mercury [49,74,75], and essential but toxic copper [75,78]. However, the direct effect of these ubiquitous chemicals on the cardiovascular system has not been extensively covered in reviews and is usually described in experimental articles. Furthermore, there are no comprehensive reviews and only limited information regarding the direct effect of these divalent cations on cardiac or skeletal muscle proteins.

### 2.2. Competition of Divalent Cations in the Body

Toxic cations such as cadmium, lead, and mercury can compete with other physiologically relevant divalent cations for entry into cells and in various biochemical reactions in the body. For example, cadmium absorption and accumulation may increase with zinc deficiency as cadmium can replace zinc in biochemical reactions [79,80,81,82,83]. Cadmium is also known to develop toxicity by competing with essential metal cations including calcium [42,84], iron, copper, and manganese for entry pathways [85]. Toxic cations can also deplete other important nutrients in the body, such as cadmium’s ability to deplete selenium, which is a crucial antioxidant and cofactor for various enzymes [86]. On the other hand, selenium is found to diminish cadmium cardiotoxicity [87]. Molecules specialized in the handling of alkaline earth (e.g., Mg^2+^, Ca^2+^) and transition metal ions (e.g., Zn^2+^, Cu^2+^, Fe^2+^) may be particularly sensitive to the presence of Cd^2+^ because they enclose cationic sites to which the toxic metal can bind [88].

Pb^2+^ can substitute calcium in calcium-binding proteins critical for heart function due to its similarity to Ca^2+^ [40,89,90,91]. Similarly, Pb^2+^ can replace other divalent metals such as zinc in zinc-finger proteins [92,93]. Lead can potentially lead to magnesium deficiency by inhibiting its absorption, which, in turn, may aggravate the negative effects of lead on the human body [94].

Both lead and mercury are powerful oxidizing agents in their +2 cationic state, with the ability to interfere significantly with processes that require specific divalent cations [40]. For example, mercury can interfere with Mg^2+^-binding sites because of their similar enthalpy energy dehydration values [40]. Interestingly, both toxic mercury and cadmium could have a competitive relationship in the accumulation in the myocardium [16].

Overall, competition between cations in the body is a vast topic that encompasses a range of divalent and trivalent ions that can compete for metal-binding structures in various tissues. For instance, Zn^2+^, Cu^2+^, Cd^2+^, and Hg^2+^ could compete for metal-binding sites of cardiac submitochondrial fragments [95]. Calcium can be replaced by strontium in various intracellular processes due to their high level of similarity [96]. Trivalent ions, e.g., Al^3+^ could also compete with Mn^2+^ and Mg^2+^ for binding sites due to their similarly small size [97,98,99].

In summary, understanding the competition between divalent and trivalent cations in the body is crucial for identifying potential strategies for preventing or treating cation-induced toxicity. Herewith, it is important to focus on their potential targets in the myocardium, as the known competition of two specific divalent cations in other tissues may be impossible in myocardium due to the absence of ways of entering for one or both cations in cardiac cells.

### 2.3. Entry Pathways for Divalent Cations into Cardiomyocytes

Various divalent cations interact with specific parts of the channels to affect their function [73]. Essential cations like calcium, magnesium, zinc, copper, etc. usually have their specific transporters or ion channels to enter cardiomyocytes [100]. For example, Mg^2+^ primarily enters cardiomyocytes through MgtE (SLC41A1) [101] and ACDP2 [102], and to a lesser extent through SLC41A2 [103], MMgT1, and MMgT2 [104].

Non-essential cations such as cadmium, lead, and mercury do not have specific ion channels or transport proteins on the cell membrane. Instead, they can utilize existing transport pathways to enter the cells [40,100]. For example, P1B-ATPases, which are transporters for cations via the membrane, are involved in the transport of Mn^2+^, Co^2+^, Cu^2+^, Ag^+^, Zn^2+^, Cd^2+^, Hg^2+^, and Pb^2+^, and may also play a role in Fe^2+^ and Ni^2+^ transport [105,106]. On the other hand, toxic metals like Pb, Cd, and Hg can enter tissues through multiple mechanisms due to ionic mimicry [107].

Cadmium has no specific ion channels or transport proteins on the cell membrane, and utilizes the transport pathways for calcium, zinc, iron, copper, and magnesium [60]. Cd^2+^ can enter cardiomyocytes through voltage-dependent Ca^2+^ channels [59,60,86], and inhibit or block the L-type Ca channel current in rabbit [108] and rat [109] ventricular myocytes. Detailed reviews devoted to cadmium entry through Ca^2+^ channels, transporters, receptors [85], and multiple sarcolemmal pathways, including Zn transporters [110], were published recently.

Lead ions can enter cardiomyocytes through various mechanisms, including Cav1.2 calcium channels [40], zinc transporters, anion exchangers, and non-voltage-dependent pathways [40]. Interestingly, currents through the Cav1.2 channels are smaller in the presence of Pb^2+^, but they last longer compared to those carried only by Ca^2+^ [40,63], similar to what has been described for inactivation by Ba^2+^ [111]. Moreover, Pb^2+^ can enter the cell through Trp channels with Ca^2+^-permeant pores that are known to be secondary entry pathways for divalent cations, in addition to other transport proteins like the Na^+^—Ca^2+^ exchanger, SOC channels, Zn^2+^ and other divalent metal transporters, pH-sensitive transporters, and aquaporin channels [40]. For details, see the review devoted to lead and mercury effects on voltage-gated calcium channel functioning [112].

Mercury ions can enter cells through various mechanisms, including ABC-type transporters and amino acid transporters and organic anion transporters. Since Hg^2+^ has a large ionic radius, it is unlikely that it enters the cardiomyocytes through Cav1.2 channels and it is more likely that it just blocks them. Although mercury may not enter through Cav1.2 channels [40], it potentially can enter cardiomyocytes through several pathways, including calcium transporters. Additionally, mercury ions can also enter cells via transporters for other divalent cations such as Zn^2+^ and Cu^2+^. It is worth noting that inorganic mercury has a low lipophilicity and thus has a limited ability to cross cell membranes [113].

For other non-essential cations, it is also possible to enter the cells. For example, nickel can enter cells through divalent metal transporters and inhibit mitochondrial function, leading to oxidative stress and DNA damage [100]. Cobalt can enter cells through calcium channels and disrupt mitochondrial function, leading to oxidative stress and apoptosis; cobalt excess can also cause severe cardiomyopathy [114,115]. The cardiac ryanodine receptor provides a suitable pathway for the rapid transport of Zn^2+^ [116], and the sarcoplasmic reticulum in cardiac muscle is suggested to act as a dynamic storage for Zn^2+^ release and reuptake [116]. Cobalt is a harmful cation that can infiltrate cells via calcium channels and interfere with mitochondrial function. This can result in oxidative stress and apoptosis, ultimately leading to severe cardiomyopathy.

**Figure 1:**
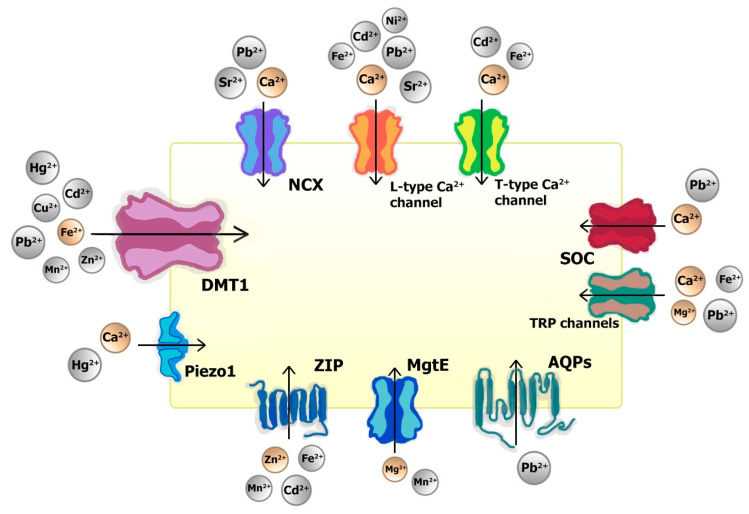
Transport pathways for divalent cations in cardiomyocytes. The native divalent cations for the channels depicted in brown, non-native divalent cations that have been confirmed to enter cardiomyocytes through the channels indicated as grey. The size of the cations in the image corresponds to their effective ionic radius.

Non-essential and non-native essential cations can use multiple transport processes to enter various cells. Most of these transport pathways have been confirmed to allow various cations to enter cardiomyocytes (Figure 1). For example, L-type calcium channels are a suitable entryway for Sr^2+^ [96], Fe^2+^ [117], Ni^2+^ [100], Cd^2+^ [118], and Pb^2+^ [40]; T-type calcium channels are suitable for Fe^2+^ [117] and Cd^2+^ [118]; store-operated channels (SOC) for Pb^2+^ [40]; transient receptor potential (TRP) channels for Fe^2+^ [117] and Pb^2+^ [40]; Piezo type mechanosensitive ion channel component 1 (Piezo1) for Hg^2+^ [119]; sodium-calcium exchanger (NCX) for Sr^2+^ [96] and Pb^2+^ [40]. Aquaporins could be used by Pb^2+^ [40]; magnesium transporters E (MgtE) by Mn^2+^ [101]; zinc-regulated, iron-regulated transporter-like proteins (ZIP) by Mn^2+^ [120], Fe^2+^ [117], and Cd^2+^ [120]; divalent metal transporter 1 (DMT1) by Mn^2+^ [121], Zn^2+^ [121], Cu^2+^ [122], Cd^2+^ [123], Pb^2+^ [40], and Hg^2+^ [124].

Once inside the cell, divalent cations such as cadmium [59,107,125,126], lead [125,126], and mercury [49,64,107] ions can bind efficiently to sulfhydryl groups (-SH) of proteins. This interaction of cations with thiol and other groups is a crucial factor in understanding the toxic effects of these cations on the body. These toxic cations can target important sites such as the calcium- and magnesium-binding sites of troponin C, the ventricular and atrial myosin regulatory light chains, and the ATP-binding pocket of myosin, leading to disruptions in cellular processes and potentially harmful cardiac effects.

### 2.4. Toxic Effects of Different Concentrations of Divalent Cations in Cells

Numerous studies have confirmed the ability of heart tissue to accumulate various divalent cations (see Section 2.1). However, the fraction of accumulation of cations specifically in cardiomyocytes remains unclear, while the heart tissue is composed of different cell types, including cardiomyocytes, cardiac fibroblasts, smooth muscle cells, endothelial cells, etc. [127]. The range of concentrations of these cations in cardiomyocytes are unknown for most cations, and therefore the evaluation of the potential toxic effect connected with their concentrations is difficult.

It could be proposed that highly toxic xenobiotic cations such as Pb^2+^, Cd^2+^, and Hg^2+^ could have an adverse effect on the heart at any concentration within cardiomyocytes. For example, the addition of Pb up to 100 μM has been shown to have negative cardioinotropic effects on cardiomyocytes [63]. Similarly, the addition of 1 mM of Cd inhibited the time-dependent hyperpolarization-activated inward current on pulmonary vein and left atrial cardiomyocytes [128]. Heightened accumulation of mercury in the myocardium has been observed in idiopathic dilated cardiomyopathy (22-fold) and secondary forms of cardiac dysfunction (3-fold) patients [15].

While free Cu is absent inside cells [129], some essential divalent cations have a certain concentration in normal conditions. For example, intracellular rest Ca^2+^ is ~0.0001 mM/L and extracellular rest ~1.2 mM/L [130]. In toxic conditions, such as malignant hyperthermia, excessive calcium release from the sarcoplasmic reticulum leads to disturbances in intracellular Ca^2+^ homeostasis, uncontrolled skeletal muscle hypermetabolism, and arrhythmias [131,132]. The cytosolic free magnesium ion concentration is 0.85 ± 0.1 mM, but it can significantly increase to a level of 2.1 ± 0.4 mM under ischemia [133]. Another study has confirmed an increase in free intracellular Mg^2+^ concentrations under ischemia [134]. However, in the myocardial tissue of men with sudden death from myocardial infarction, the Mg^2+^ concentration is decreased [135].

To summarize, further research is needed to better understand the concentration range of various cations in cardiomyocytes, a topic which is still is largely unknown, which makes it challenging to assess their potential toxic effects.

## 3. EF-Hand Protein Family and Divalent Cations

The EF-hand family are calcium-binding proteins with a structural motif called the EF-hand, which participate in various cellular processes, including muscle contraction [136]. The EF-hand protein family has been studied extensively, and Snyder et al. [137] have concluded that the binding of metal ions to the EF-hand motif is dependent on their charge and size [137]. The EF-hand binding sites are specifically designed to bind highly charged cations, resulting in low affinities for monovalent metals such as Na^+^ and K^+^. Therefore, these metals are not included in our research focus, despite the fact that myocardium is known to accumulate various chemicals, including monovalent ones [27]. The size of the cavity of binding sites is optimized for divalent metals that have effective ionic radii similar to Ca^2+^, i.e., optimal binding occurs at an ionic radius between those of Mg^2+^ (0.81 A) and Ca^2+^ (1.06 A) [137]. Additionally, trivalent metal ions of the group IIIA and the lanthanides also exhibit size-dependent affinities, with an optimal effective ionic radius between those of Sc^3+^ (0.81 A) and Yb^3+^ (0.925 A) [137].

Calmodulin plays a crucial role in regulating myosin light chain kinase activity in smooth muscles and, being a member of the EF-hand family, has calcium-binding sites [138]. It has been shown that Ca^2+^ can be substituted by bi- and trivalent cations with similar ionic radii viz. Zn^2+^, Mn^2+^, Cd^2+^, Sr^2+^, Pb^2+^, Cr^2+^, Tb^3+^, Sm^3+^, and La^+^ [84,139], altering the conformation of calmodulin. Sr^2+^, Pb^2+^, Cd^2+^, and Cr^2+^ can stimulate the activity of calmodulin to varying degrees, from 60% to 90% [138,139]. Sr^2+^ is also known to bind to α-parvalbumin, which is another member of the EF-hand family [140]. On the other hand, Na^+^, Mg^2+^, Ba^2+^, and Ni^2+^ had no distinct effect on calmodulin [84]. Data on the affinity of Al^3+^ and Hg^2+^ to calmodulin are contradictory [84,139,141], though there is an opinion that Zn^2+^, Al^3+^, and Hg^2+^ do not affect calmodulin activity at concentrations up to 1 mM [139]. It is noteworthy that various cations may exhibit different degrees of affinity towards the distinct binding sites of calmodulin [39], with the lead having a higher affinity for calmodulin binding sites than other metals [142], especially in the N-terminal domain where Pb^2+^ binds with an 8-fold higher affinity than Ca^2+^ [90].

Concerning proteins affected by lead, de Souza et al. [142] described many such proteins and proposed that their number is probably much higher. This hypothesis could be proposed for proteins of the EF-hand family and other cations. Such ionic displacement (e.g., ionic mimicry) or opportunistic binding of cations to proteins [90], such as with the EF-hand family, could underlie the mechanisms of toxicity.

### 3.1. Troponin C

Troponin C is a subunit of cardiac and skeletal troponin and, like calmodulin, it is a member of the EF-hand family, which has calcium-binding sites [143]. In both skeletal and cardiac troponin C, there are Ca^2+^ binding sites, two of which are high-affinity sites, which can also bind other divalent cations such as Mg^2+^ and Cd^2+^ [143]. Troponin C also has low-affinity Ca^2+^-specific sites (2 sites for skeletal TnC and 1 site for cardiac TnC), which are highly selective to Ca^2+^ under a normal physiological concentration of Mg^2+^ [143]. Previous studies have shown that Ca^2+^ could be substituted by Cd^2+^ (72% of calcium ions were substituted), Sr^2+^ (57%), Pb^2+^ (45%), and Mn^2+^ (35%), Co^2+^ (12%), Ni^2+^ (10%), Ba^2+^ (10%), Mg^2+^ (8%), and Zn^2+^ (6%) ions with a concentration 0.1 mM. The affinity of divalent cations for the receptor site of troponin C is closely related to their ionic radius and its proximity to the ionic radius of Ca^2+^.

Trivalent lanthanide ions such as La^3+^, Ce^3+^, Nd^3+^, Sm^3+^, and Dy^3+^ can partially displace about 25–35% of the bound Ca^2+^ from troponin C at a concentration of 0.02 mM, but their affinity for the receptor site is lower than that of Ca^2+^ despite their similarity to Ca^2+^ in size and chemical properties. To compare, the same concentrations of Cd^2+^ and Sr^2+^ could displace 40 and 29% of the bound Ca^2+^, respectively [144]. Pb^2+^ and Cd^2+^ can bind to skeletal muscle troponin C and activate myosin ATPase activity at a low concentration, but significantly decrease ATPase activity at concentrations higher than 100 µM [42]. Interestingly, Sr^2+^ can activate the contractile process in diaphragm fibres, and the response is dependent on the composition of TnC isoforms, with the slow isoform conferring on average a 7-fold greater sensitivity to Sr^2+^ than the fast isoform [145].

### 3.2. Regulatory Light Chain

Cardiac myosin filaments are directly regulated by calcium ions, as reported by Ma et al. [146]. Currently, two substructures of myosin that are capable of binding calcium ions are confirmed: the ATP-binding pocket and the regulatory light chain (RLC). The myosin RLC belongs to the EF-hand family and contains the EF-hand domain responsible for binding calcium and magnesium cations [147]. Under relaxed conditions, the myosin RLC EF-hand domains have been observed to bind magnesium (Mg^2+^), but as the concentration of calcium ions increases during muscle contraction, they become increasingly occupied by Ca^2+^ [148]. At the same time, the elevated free Mg^2+^ concentration reduces the Ca^2+^ sensitivity of skeletal myosin and this effect could be associated with the influence of the RLC [149].

The function of cation-binding sites in muscle contraction is important, but not fully understood [148]. Optimal binding to the EF-hand occurs at an ionic radius close to Mg^2+^ and Ca^2+^. However, the affinity of the EF-hand-like Ca^2+^ binding sites [97,150], including those of smooth muscle RLC [151], to cations can be significantly altered by amino acid substitutions. For instance, Da Silva et al. [151] demonstrated that amino acid substitutions could alter the affinity of the EF-hand in smooth muscle RLC to divalent ions (Mg^2+^ or Ca^2+^). This effect may be important in mutations that cause alterations in the amino acid sequence. Moreover, post-translational modification hypothetically could influence the affinity of proteins to divalent cations. For α-synuclein, it has been shown that phosphorylation in very close proximity to the putative metal binding sites, could increase the binding affinity of Cu(II), Pb(II), and Fe(II) [152]. However, it remains unclear whether this is also true for EF-hand proteins.

It is highly possible that various cations, including toxic Pb^2+^, Cd^2+^, and Hg^2+^, may compete with Ca^2+^ not only for the calcium-binding sites of calmodulin [138] and skeletal troponin C [42] as described above, but also for other members of the EF-hand family, such as cardiac troponin C and atrial and ventricular myosin regulatory light chains.

## 4. The Myosin ATP-Binding Pocket

The myosin ATP-binding pocket binds both ATP and Mg^2+^ (MgATP) to coordinate ATP hydrolysis for force generation and muscle contraction [153]. While MgATP is the natural substrate [154], myosin is not a specific ATPase, and the myosin ATP-binding pocket can bind other divalent cations, such as Ca^2+^ [153,154,155], and Mn^2+^ [153], which support myosin ATPase activity (Table 1).

The concentration of CaATP in the sarcomere reaches 64–71 μM [156], which is about 1% of all available ATP [155]. The rate of the basal CaATPase activity is higher than that of MgATPase [153,154,155,157], and actin-activated CaATPase activity is also higher than that of MgATPase [155,157], or does not have a significant difference [153,154]. The rate of ATP binding is higher in the presence of Ca^2+^ than in the presence of Mg^2+^ [155], and at the same time, CaATP dissociates actomyosin 3–4 times slower than MgATP [154,155,157], increasing the time of strong actomyosin binding. The authors pointed out a higher actin affinity when S1-CaADP- Pi is converted to S1-CaADP, compared to the magnesium case [154], and that CaATP binds tighter to myosin than MgATP [155]. It was shown that the isometric force produced by muscle fibres when CaATP was the substrate is only 20% of the value obtained with MgATP [154]. Later, it was proposed that myosin readily produces futile strokes with CaATP [155]. At the same time, the gliding assay indicated that myosin did not propel F-actin in the presence of Ca^2+^ ions [153]. In healthy muscles, contraction occurs regardless of the small amount of CaATP that is always present in the muscle [155,156]. However, Ge et al., proposed that an increase of Ca^2+^ during disturbed calcium homeostasis in the muscle will increase the probability of finding CaATP [155].

In the presence of physiologically essential Mn^2+^, the rate of basal [153,157,158] and actin-activated [153,159,160] MnATPase activity is higher than that of MgATPase. However, there was no statistically significant F-actin stimulation for MnATPase [153], and no alteration in the mechanical characteristics of skeletal fibres [160]. In the presence of Mn^2+^, the sliding velocity of F-actin over myosin was inhibited approximately 3-fold compared to Mg^2+^ [153]. The rate of movement of the myosin lever arm and phosphate release in MnATPase cycles are significantly accelerated compared to the MgATPase cycle [157]. Walker et al. concluded that a significant proportion of myosin might undergo the power stroke and phosphate release before reattaching to the F-actin filament. As a result, there is an increase in the probability of futile power strokes that could explain the reduced motility observed for myosin in the presence of Mn^2+^ compared to Mg^2+^ [153].

If essential cations can significantly influence the ATPase activity, xenobiotics with a high degree of probability may have a similar effect. For instance, acute lead exposure could influence myocardial contractility through alterations in the ATPase activity of myosin [62]. Specifically, exposure to 8 μM lead acetate was shown to increase the ATPase activity of left-ventricular myosin by 30% [161]. However, exposure to 100 μM lead acetate has been shown to reduce ATPase activity of right-ventricular myosin by 26% [62]. This contradiction could be a result of different doses of lead or different ventricles from which the myosin was extracted. The first assumption is more possible, as myosin of both ventricles have the same structure, and may differ only in the ratio of myosin heavy chain isoforms [162]. Chao et al. have shown that myofibrillar PbATPase activity in the presence of TnC was higher than that of CaATPase at a concentration of ca. 100 µM and decreased at higher concentrations. Without TnC, myofibrillar PbATPase was comparable to that of CaATPase at concentrations lower than 75 µM and decreased at higher concentrations [42].

Similar results were shown for cadmium: myofibrillar CdATPase activity in the presence of TnC was lower than that of CaATPase. Without TnC, myofibrillar PbATPase was comparable to that of CaATPase at concentrations lower than 75 µM and increased at higher concentrations [42].

Extracellular Hg^2+^ significantly reduces the contraction of isolated guinea-pig cardiomyocytes [40] and inhibits myosin ATPase activity. Myosin ATPase activity was shown to be completely blocked with 1 μM Hg^2+^ and reduced by 50% with 0.15 μM Hg^2+^ [65]. Later, similar results were shown, i.e., HgCl_2_ reduced the activity of myosin ATPase in a dose-dependent manner, beginning from small concentrations of 50 nM, and achieved about a 45% reduction in activity following exposure to 100 nM HgCl_2_ [64].

Many other divalent cations are also able to support myosin ATPase activity in experimental conditions. For instance, Fe^2+^, Mn^2+^, Ni^2+^, and Co^2+^ support the ATPase activity of S1-myosin in the absence of actin, and this activity increases with the increasing ionic radius of the metal (Me) ions [159]. These metal ions also support actin activation; however, there is no strong correlation between actin-activated MeATPase activity and their ionic radii [159]. At the same time, NiATP does not support muscle fibre contraction [160]. Zn^2+^ [163] and Cu^2+^ [67] significantly inhibit ATPase activity, while Al has no effect [163]. Wherein, Sr^2+^ has been found to activate actomyosin ATPase and has often been used as a Ca^2+^ surrogate to regulate muscle contraction in experimental conditions [4].

In a normal actin-myosin interaction, Mg^2+^ stimulates myosin ATPase activity. However, other divalent cations can significantly affect the kinetics of myosin. The specific cation appears to play a critical role in this effect. Physiologically relevant cations (Ca^2+^, Mn^2+^) could intervene in the ATPase activity, and low concentrations of the toxic and xenobiotic metals, e.g., Pb^2+^, Hg^2+^, Cd^2+^ inhibit the activity of myosin ATPase, suggesting a strong toxic effect. Previous studies have shown a linear relationship between the ionic radius of Ca^2+^, Mg^2+^, and Mn^2+^ and the respective basal and actin-activated ATPase activities in the presence of these cations [157]. The larger the ionic radius, the higher the myosin ATPase activity. However, it should be checked for more cations, which are shown to have myosin ATPase activity e.g., Pb, Cd, Hg etc. Interestingly, actin activation of myosin ATPase does not depend on the metal cation, despite the cation-specific kinetics of nucleotide binding and dissociation. The rate-limiting step of myosin ATPase activity depends on the metal cation, with the rate of the recovery stroke and reverse recovery stroke being directly proportional to the ionic radius of the cation. The rate of nucleotide release from myosin and actomyosin and ATP binding to actomyosin depends on the cation coordination number [157].

**Table 1:** Influence of divalent cations on myosin ATPase activity.

Cation	Compound	Concentration	Change of Basal ATPase Activity, % *	Change of Actin-Activated ATPase Activity, % *	Velocity in Motility (Gliding) Assay, % *	Object	Reference
Ca	CaCl_2_	1 µM	−10			Rabbit fast skeletal muscle myofibrils	Chao, 1990 [42]
		10 µM	+305				
		50 µM	+350				
		100 µM	+350				
		1 mM	+328				
		1 mM	+655	−52		Rabbit fast skeletal muscle myosin S1 fragment	Peyser, 1996 [159]
		2 mM			No motility	Dictyostelium discoideum myosin II	Walker, 2021 [153]
		5 mM	+1450	+475			
			+4333	+516		Rabbit fast skeletal muscle myosin	Ge, 2019 [155]
			+3544	−16			Polosukhina, 2000 [154]
Mn	MnCl_2_	1 mM	+383	+12		Myosin S1 fragment back and leg muscles of rabbit	Peyser, 1996 [159]
		2 mM			−63	Dictyostelium discoideum myosin II	Walker, 2021 [153]
		5 mM	+4900	+1050		Dictyostelium discoideum myosin II	Walker, 2021 [153]
		6 mM		+32		Rabbit fast skeletal muscle myosin S1 fragment	Burton, 2005 [160]
Pb	Pb(C_2_H_3_O_2_)_2_	8 µM	+30			Rat left ventricle myosin	Fioresi, 2013 [161]
		100 µM	−26			Rat right ventricle myosin	Vassallo, 2008 [62]
	PbCl_2_	1 µM	−1			Rabbit fast skeletal muscle myofibrils	Chao, 1990 [42]
		10 µM	+215				
		50 µM	+395				
		100 µM	+373				
		1 mM	+58				
Cd	CdCl_2_	1 µM	−15			Rabbit fast skeletal muscle myofibrils	Chao, 1990 [42]
		10 µM	+170				
		50 µM	+340				
		100 µM	+305				
		1 mM	No activity				
Hg	HgCl_2_	50 nM	−20			Rat left ventricle myosin	Moreira, 2003 [64]
		100 nM	−40				
		150 nM	−50				Vassalo, 1999 [65]
		200 nM	−55				Moreira, 2003 [64]
		300 nM	−70				
		400 nM	−80				
Cu	CuCl_2_	10 μg/mL	−67			Rat left ventricle myosin	Filetti, 2018 [67]
Ni	NiCl_2_	1 mM	−43	−76		Rabbit fast skeletal muscle myosin S1 fragment	Peyser, 1996 [159]
	Ni(CH_3_CO_2_)_2_	5 mM		−63		Rabbit fast skeletal muscle myosin S1 fragment	Burton, 2005 [160]
Zn	ZnSO_4_	100 µM	−40			Rabbit fast skeletal muscle myosin	Jones, 1997 [163]
		250 µM	−75				
		500 µM	−85				
		1 mM	−90				
Al	AlCl_3_	100 µM	No effect			Rabbit fast skeletal muscle myosin	Jones, 1997 [163]
		250 µM	+10				
		500 µM	+10				
		1 mM	No effect				
	AlC_6_H_5_O	100 µM	No effect			Rabbit fast skeletal muscle myosin	Jones, 1997 [163]
		250 µM	No effect				
		500 µM	No effect				
		1 mM	No effect				
Fe	FeCl_2_	1 mM	+360	−45		Rabbit fast skeletal muscle myosin S1 fragment	Peyser, 1996 [159]
Co	CoCl_2_	1 mM	+262	−27		Rabbit fast skeletal muscle myosin S1 fragment	Peyser, 1996 [159]

* Percentage change of described ATPase compared to native or experimental MgATPase activity.

It is worth noting that divalent cations have been shown to influence the ATPase activity of various motor proteins, including kinesin [164], non-muscle [165], and muscle [154,155] myosin. Walker et al. pointed out that in kinesin, microtubule gliding velocities, as well as basal and microtubule-stimulated ATPase rates, were similar with either Mn^2+^ or Mg^2+^ [164], unlike in myosin where the basal ATPase activity was stimulated by MnATP but not by MgATP [165]. Apparently, different motor proteins and isoforms of myosin may respond differently to the same cations due to differences in their structure. Therefore, it is essential to investigate how various cations can affect different motor proteins. Moreover, it is important to consider the possibility that ATPase activity may be influenced not only by cation interactions with the myosin ATP-binding pocket but also by metal binding to the RLC or other unknown mechanisms. Further investigation is necessary to fully understand the impact of metal binding on the RLC and its potential effect on ATPase activity.

## 5. Materials and Methods

This review was prepared following the PRISMA statement guidelines [166]. Using databases MEDLINE (via PubMed) and Google Scholar, we searched for articles that met specific criteria:

(1) In the article title, at least one of the following words was found: “heart OR cardiomyocytes OR cardiotoxicity OR myocardial OR cardiac OR cardiovascular OR cardiomyopathy”.
(2) In the found article, the chemical symbol, English, or Latin name of the divalent cation must be present in the article body. Namely [167], beryllium (Be^2+^), magnesium (Mg^2+^), calcium (Ca^2+^), strontium (Sr^2+^), barium (Ba^2+^), radium (Ra^2+^), vanadium (Va^2+^), chromium (Cr^2+^), manganese (Mn^2+^), iron/ferrum (Fe^2+^), ruthenium (Ru^2+^), osmium (Os^2+^), cobalt (Co^2+^), rhodium (Rh^2+^), nickel (Ni^2+^), palladium (Pd^2+^), platinum (Pt^2+^), copper/cuprum (Cu^2+^), zinc (Zn^2+^), cadmium (Cd^2+^), mercury (Hg^2+^), tin/stannum (Sn^2+^), lead/plumbum (Pb^2+^), polonium (Po^2+^). Divalent lanthanides that are known in the solid state but are unstable in water: neodymium (Nd^2+^), samarium (Sm^2+^), europium (Eu^2+^), dysprosium (Dy^2+^), Thulium (Tm^2+^), ytterbium (Yb^2+^). Divalent actinides (all artificially made): americium (Am^2+^), californium (Cf^2+^), einsteinium (Es^2+^), fermium (Fm^2+^), mendelevium (Md^2+^), nobelium (No^2+^).
(3) In the found article, at least one of the words must be present in the text “transport”, “channel”, “accumulation”, “cardiotoxicity”, or “intoxication”. Additional search and selection were also made on the exact phrase according to the template “divalent cation + key word”, e.g., “copper cardiotoxicity”. All publications were screened based on their title and abstract to assess their relevance and eligibility, with special attention given to the known or proposed influence of divalent cations on the contractile or regulatory muscle proteins. More than 170 papers were carefully read and processed, and ca. 135 articles devoted to cations were utilized for the manuscript draft, which was revised several times.

The curation of text was conducted by dint of Artificial Intelligence (AI) Language Model ChatGPT 3.5 using an enquiry “AI, improve the following text with special attention to English grammar, punctuation, and grammatical form of time”. The AI made minor improvements to the text, but the authors retained discretion over word choice and final correction. The total time spent working on the manuscript took more than 8000 min, excluding the search, selection, and reading of articles. After revisions, a final version of the manuscript was prepared and approved by all the authors.

## 6. Conclusions

Heart contraction is a highly coordinated process that involves the interplay of multiple molecules, including calcium- and magnesium-binding protein structures. Essential and xenobiotic divalent cations can accumulate in the myocardium, enter cardiac cells, and possibly bind to proteins, altering their functions.

The EF-hand family proteins have binding sites optimized for highly charged cations that have effective ionic radii close to that of Mg^2+^ and Ca^2+^. However, only a few members of the EF-hand family viz. calmodulin, α-parvalbumin, skeletal troponin C, and smooth muscle RLC were investigated concerning their ability to bind various divalent cations.

Calmodulin alters its conformation when at binding sites where Ca^2+^ has been substituted by bi- or trivalent cations with similar ionic radii viz. Cd^2+^, Sr^2+^, Pb^2+^, Cr^2+,^ Zn^2+^, Mn^2+^, Tb^3+^, Sm^3+^, and La^+^. Data on the affinity of Al^3+^ and Hg^2+^ to calmodulin are contradictory. Cd^2+^, Sr^2+^, Pb^2+^, Cr^2+^ stimulate the calmodulin activity to varying degrees. Zn^2+^, Al^3+^, and Hg^2+^ do not affect calmodulin activity at concentrations up to 1 mM. Na^+^, Mg^2+^, Ba^2+^, and Ni^2+^ had no distinct effect on calmodulin. α-parvalbumin, which is a canonical calcium-binding protein, was shown to bind Sr^2+^. In skeletal Troponin C, Cd^2+^, Sr^2+^, Pb^2+^, Mn^2+^, Co^2+^, Ni^2+^, Ba^2+^, Mg^2+^, Zn^2+^, and trivalent lanthanides such as La^3+^, Ce^3+^, Nd^3+^, Sm^3+^, and Dy^3+^ can substitute for Ca^2+^. Smooth muscle RLC was shown to significantly alter affinity to divalent cations, i.e., Ca^2+^ and Mg^2+^, after amino acid substitutions.

Thus, various cations, including essential Zn^2+^, Cu^2+^, etc., and toxic Pb^2+^, Cd^2+^, and Hg^2+^, may compete with Ca^2+^ for the calcium-binding sites of EF-hand proteins, altering their functions. Therefore, it is necessary to study whether cardiac troponin C and atrial and ventricular myosin regulatory light chains could be potential targets for these cations.

As myosin ATPase is not specific, many cations, including Ca^2+^, Fe^2+^, Mn^2+^, Ni^2+^, Sr^2+^, and Co^2+^, could support its ATPase activity. At the same time, some other cations such as Zn^2+^ and Cu^2^ significantly inhibit ATPase activity. Therefore, further investigation is necessary to fully understand the possibility of cations binding by a myosin ATP-binding pocket. It is important to consider the possibility that ATPase activity may be influenced not only by cation interactions with the myosin ATP-binding pocket but also by metal binding to the RLC.

The main question is whether cations could influence myosin and troponin kinetics in real conditions. In healthy muscles, contraction occurs regardless of the small amount of e.g., CaATP that is always present in the muscle. However, in certain disturbances of cation homeostasis, concentration of physiologically relevant divalent ions can be increased, significantly affecting the kinetics of the interaction between myosin and actomyosin. For instance, Ca^2+^ can be increased and CaATPase activity may contribute to observed muscle rigidity and enhanced muscle thermogenesis as in malignant hyperthermia, or Cu^2+^ can significantly increase in Wilson disease [168], with uninvestigated possible effects. The potential effect apparently depends on the ionic radius and charge of the specific cation, its concentration in the cell, and the specific isoform of protein. In addition, the affinity of EF-hand-like Ca^2+^ binding sites or other targets for divalent ions may be significantly altered by amino acid substitutions or post-translational modification.

Further investigation is necessary to fully understand the mechanisms of cation entry into cells, their competition, possible targets, affinity to these targets in different cation concentrations, and their influence on the mechanical characteristics of cardiac contractile and regulatory proteins. It is important to thoroughly study both essential and xenobiotic cations, with a priority on toxic cations, while not omitting trivalent cations. We hope that our review will help to provide direction for further investigations.

## Data Availability

No new data were created or analyzed in this study.
